# Influence of Traffic Activity on Heavy Metal Concentrations of Roadside Farmland Soil in Mountainous Areas

**DOI:** 10.3390/ijerph9051715

**Published:** 2012-05-07

**Authors:** Fan Zhang, Xuedong Yan, Chen Zeng, Man Zhang, Suraj Shrestha, Lochan Prasad Devkota, Tandong Yao

**Affiliations:** 1 Key Laboratory of Tibetan Environment Changes and Land Surface Processes, Institute of Tibetan Plateau, Chinese Academy of Sciences, Beijing 100080, China; Email: zhangfan@itpcas.ac.cn (F.Z.); zengchen@itpcas.ac.cn (C.Z.); 10125479@bjtu.edu.cn (M.Z.); yaotandong@itpcas.ac.cn (T.Y.); 2 MOE Key Laboratory for Urban Transportation Complex Systems Theory and Technology, Beijing Jiaotong University, Beijing 100044, China; 3 State Key Laboratory of Rail Traffic Control and Safety, Beijing Jiaotong University, Beijing 100044, China; 4 Central Department of Hydrology and Meteorology, Tribhuvan University, Kathmandu 44618, Nepal; Email: theshrestha.amigo@gmail.com (S.S.); devkotalp@hotmail.com (L.P.D.)

**Keywords:** heavy metal (Cu, Zn, Cd, and Pb), roadside farmland soil, mountainous highway, Nepal

## Abstract

Emission of heavy metals from traffic activities is an important pollution source to roadside farmland ecosystems. However, little previous research has been conducted to investigate heavy metal concentrations of roadside farmland soil in mountainous areas. Owing to more complex roadside environments and more intense driving conditions on mountainous highways, heavy metal accumulation and distribution patterns in farmland soil due to traffic activity could be different from those on plain highways. In this study, design factors including altitude, roadside distance, terrain, and tree protection were considered to analyze their influences on Cu, Zn, Cd, and Pb concentrations in farmland soils along a mountain highway around Kathmandu, Nepal. On average, the concentrations of Cu, Zn, Cd, and Pb at the sampling sites are lower than the tolerable levels. Correspondingly, pollution index analysis does not show serious roadside pollution owing to traffic emissions either. However, some maximum Zn, Cd, and Pb concentrations are close to or higher than the tolerable level, indicating that although average accumulations of heavy metals pose no hazard in the region, some spots with peak concentrations may be severely polluted. The correlation analysis indicates that either Cu or Cd content is found to be significantly correlated with Zn and Pb content while there is no significant correlation between Cu and Cd. The pattern can be reasonably explained by the vehicular heavy metal emission mechanisms, which proves the heavy metals’ homology of the traffic pollution source. Furthermore, the independent factors show complex interaction effects on heavy metal concentrations in the mountainous roadside soil, which indicate quite a different distribution pattern from previous studies focusing on urban roadside environments. It is found that the Pb concentration in the downgrade roadside soil is significantly lower than that in the upgrade soil while the Zn concentration in the downgrade roadside soil is marginally higher than in the upgrade soil; and the concentrations of Cu and Pb in the roadside soils with tree protection are significantly lower than those without tree protection. However, the attenuation pattern of heavy metal concentrations as a function of roadside distance within a 100 m range cannot be identified consistently.

## 1. Introduction

The risk posed by heavy metals to food safety and the environment are of great concern to governments and society in many countries. Heavy metal pollution in agricultural soils is becoming serious with the rapid industrialization and urbanization in developing countries [1]. This is a typical environmental issue due to anthropogenic activities in the countries in the Third Pole region which consists of the Tibetan Plateau and surrounding mountains. The Third Pole region covers parts of eight countries, consisting China, India, Russia, Bhutan, Myanmar, Nepal, Pakistan and Afghanistan, in which a fifth of the World’s population live [2]. Though the Third Pole region is relatively underdeveloped, it has undergone rapid economic growth and booming tourism in recent years, which have brought an increase of transportation activities and related pollution. The pressure on the unique ecosystem in the anthropogenic living areas of the Third Pole region is predictably increasing [3]. Traffic activities are one of the major sources leading to heavy metal contamination in roadside soils due to their long-term accumulation. Therefore, the local contamination resulting from transportation activities is receiving increasing attention in the Third Pole countries. 

As one of the developing counties in the Third Pole region, Nepal’s agriculture has been the mainstay of the economy, accounting for 40 percent of GDP and 60 percent of the labor force. In Nepal, overland transportation is the major transportation mode but road density is low at 11.4 km of road per 100 km^2^ and 0.71 km per 1,000 population, mainly because of the country’s complex mountainous topography and insufficient resources. Although traffic volume is generally low in the remote rural areas in Nepal, the cumulative contamination effect of long-term exposure to traffic activities cannot be neglected. More importantly, the roadside farmland soil is associated with the food chain and public health.

Heavy metals can directly harm public health by entering the body via soil and dust, dermal contact or breathing [4]. The typical elements Cd, Pb, Zn, and Cu in the roadside soils coming from traffic activity can be transported through the food chain into the human body and thus be very toxic to people. In agricultural areas, intake of heavy metals through the soil-crop system could play a predominant role in human exposure to heavy metals [5]. In general, heavy metals with high concentrations in the environment result in health problems adversely affecting the nervous, blood forming, cardiovascular, renal and reproductive systems. The consequences of heavy metal pollution include reduced intelligence, attention deficit and behavioral abnormality, as well as contribution to cardiovascular disease in adults [6]. Some trace metals (such as Cu and Zn) are harmless in small amounts, but the others (mainly Pb, As, Hg and Cd), even at extremely low concentrations, are toxic and are potential cofactors, initiators or promoters in many diseases, including increased risk of cancer [7,8]. However, it is not easy to remove heavy metals from the soils because of their irreversible immobilization within different soil components [9].

The mechanisms of heavy metal emission from vehicles consist of fuel consumption, engine oil consumption, tire wear, brake wear, and road abrasion [10,11,12]. Engine oil consumption is responsible for the largest emission for Cd, tire wear contributes the most important emission for Zn, and brake wear is the most important source of emissions for Cu and Pb [12]. Though the use of unleaded gasoline has caused a subsequent reduction in fuel emissions of Pb, it may still occur in exhaust gas and come from worn metal alloys in the engine [12]. Bitumen and mineral filler materials in asphalt road surfaces contain different heavy metal species, including Cu, Zn, Cd, and Pb [12]. Heavy metals can be transported into the roadside soils by atmospheric precipitation or road runoff [13,14].

Monitoring studies have been conducted in many cities and regions to investigate the roadside heavy metal contamination, including China’s Hong Kong [15,16,17], Beijing [18], and Shanghai [19], Mexico City [20], Turkey’s Elazig [21], England’s Yorkshire [22], Jordan’s Amman [23], Greece’s Kavala [24], *etc*. It was found that roadside heavy metal concentration is influenced by multiple factors, including traffic properties, highway characteristics, roadside terrain, roadside distance, wind direction, *etc*. 

Generally, the longer the highway usage history, the higher the concentration in the roadside soil [25], because it is positively related to traffic volume [18]. Normally, the heavy metal content in roadside soils has a belt-shaped distribution in terms of distance to road edge, decreasing exponentially with distance from road [26]. Compared to the background nature value of heavy metal content, the influential space of traffic pollution can be up to 50 m far from road but within 100 m [27,28]. In addition, most of the deposited metal particles remain in the 0–5 cm of the roadside surface soil depth [29]. The plants along roadside also have higher heavy metal content and can effectively lower the concentration of heavy metals in soil [30,31]. Few studies with factorial design analyses were focused on the heavy metal contamination in rural roadside farmland soils due to traffic activities. More importantly, no previous research was conducted to investigate the roadside soil heavy metal pollution in mountainous areas.

This research aims to investigate the influence of transportation activities on farmland soils along a highway across mountainous areas in Nepal. Corresponding to the complex geographic feature of mountain highways, a study with Multivariate Analysis of Variance (MANOVA) design was conducted to analyze the effects of highway altitude, roadside distance, roadside terrain, and tree protection on the concentrations of Cu, Zn, Cd, and Pb in the farmland soil in mountainous areas and thereby indicate potential mitigation strategies for realization of human-nature harmony in the Third Pole. 

## 2. Material and Methods

### 2.1. Site Description

**Figure 1:**
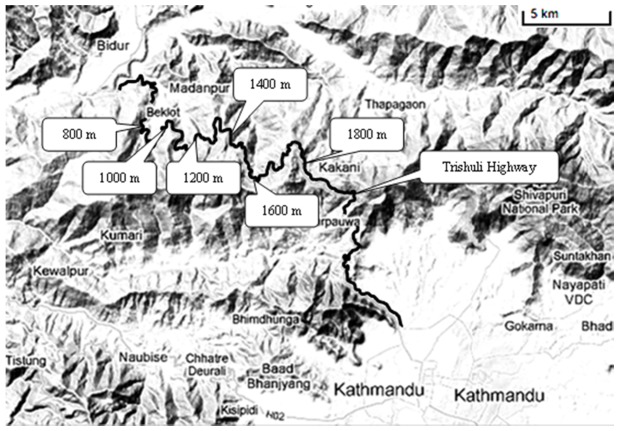
Sampling locations and altitudes along Trishuli Highway between Trishuli City and Kathmadu, Nepal.

In this study, a rural highway (Trishuli Highway) crossing a typical mountainous farmland area between Trishuli City and Kathmadu, the capital of Nepal, was selected (See Figure 1). In the area, the annual temperatures range from freezing point to over 30 °C. July, August and September are the monsoon months. The yearly average rainfall is 1,850 millimeters per year. The Trishuli Highway is an all-weather gravel/dirt (4.5 m) track with a 40 mph design speed and low traffic volume of ~1,569 vehicles per day. The vehicles are composed of 33% motorcycles, 15% cars, 27% buses, 24% trucks, and 1% tractors [32]. The highway’s altitude ranges from 730 m to 1,900 m. Due to the complex terrain of the study area, the roadside farmland is either upgrade or downgrade, and closely connected to the road edges.

### 2.2. Factorial Experiment Design

According to the characteristics of the study site in mountainous areas in the Third Pole region, the analysis followed a 6 × 5 × 2 × 2 MANOVA design for analyzing whether the independent variables Altitude, Distance, Terrain, and Tree have a statistically significant effect on the concentration distribution of four heavy metals (Cu, Zn, Cd, and Pb) in the roadside farmland soil. Table 1 provides a detailed description of the four independent variables. Altitude is defined as the true elevation above mean sea level. Six altitude levels range from 800 m to 1,800 m with increments of 200 m. Distance is defined as the distance from the soil sampling location perpendicular to the road edge. The sampling distances to the highway edge are designated as 0 m, 10 m, 30 m, 50 m, and 100 m, assuming that 100 m samples represent the background heavy metal contents. Two types of roadside farmland terrains (upgrade or downgrade) are designated, assuming that drainage direction to the road corresponding to the roadside slope direction may affect the concentration distribution of heavy metals. Since trees intermittently grow along the Trishuli Highway, whether the trees have a protection effect on the heavy metal pollution was also investigated.

**Table 1:** Description of four independent variables for MANOVA analysis.

Independent Variable	Variable Definition	Discrete Level
Altitude	Elevation above mean sea level. Six levels are designated along the Trishuli Highway.	Level 1: 800 m
		Level 2: 1,000 m
		Level 3: 1,200 m
		Level 4: 1,400 m
		Level 5: 1,600 m
		Level 6: 1,800 m
Distance	Distance from the soil sampling location perpendicular to the road edge. Five distances are designated.	Level 1: 0 m
		Level 2: 10 m
		Level 3: 30 m
		Level 4: 50 m
		Level 5: 100 m
Terrain	Roadside farmland slope direction. Upgrade or downgrade is used to describe whether the roadside farmland gradually arise or going down from the road surface.	Level 1: Upgrade—farmland elevation gradually increases from the road surface.
		Level 2: Downgrade—farmland elevation gradually decreases from the road surface.
Tree	Tree is designated to describe whether there are trees growing along the road edge or not, and test whether the trees can protect the farmland soil from metal pollution.	Level 1: Tree—Trees exist between farmland and roads and the trees’ continues distance is at least 30m along the road direction.
		Level 2: No Tree—There are no trees between farmland and road.

### 2.3. Soil Sampling

A total of 342 topsoil samples from the depth of 0–5 cm were collected under dry weather conditions along the highway at the six levels of altitudes. Table 2 lists the sample distribution cross-tabulation by the independent variables of Altitude × Distance × Tree × Terrain. It was planned to collect three samples for each cell in order to achieve a full factorial design. However, limited to the on-site conditions, the cases of 1,200 m altitude, downgrade terrain with trees could not be found; the sample was not available in the case of 1,400 m altitude, downgrade terrain without trees at 0 m distance; and the farmland soil was not available in the case of 1,600 m altitude, upgrade terrain with trees at 100 m distance.

**Figure 2:**
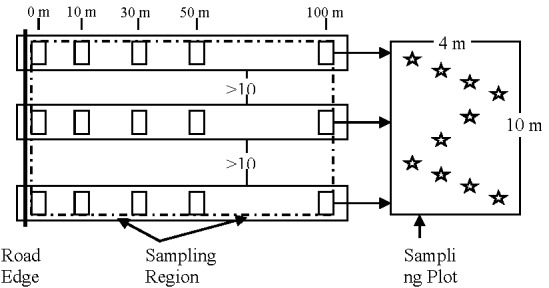
Illustration of sampling method. Three sets of samples are collected in three sampling regions at each sampling location. In each sample region, five samples are taken with five sampling distances to the highway edge. For each sample, 8–10 sub-samples were taken in an ‘S-shape’ pattern and evenly mixed.

As shown in Figure 2, at each sampling location, three sets of samples were collected in three sampling regions with spacing not less than 10 m to minimize their dependency. The sampling distances to the highway edge were designated as 0 m, 10 m, 30 m, 50 m, and 100 m. For each sample, 8–10 topsoil sub-samples were taken in an ‘S-shape’ pattern in a 10 m × 4 m plot and evenly mixed.

**Table 2:** Sample distribution cross-tabulation by Altitude × Distance × Tree × Terrain.

Grade	Tree	Distance	Altitude (m)						Total
			800	1,000	1,200	1,400	1,600	1,800	
Down grade	No tree	0 m	3	3	3	-	3	3	15
		10 m	3	3	3	3	3	3	18
		30 m	3	3	3	3	3	3	18
		50 m	3	3	3	3	3	6	21
		100 m	3	3	3	3	3	3	18
	Tree	0 m	3	3	-	3	3	3	15
		10 m	3	3	-	3	3	3	15
		30 m	3	3	-	3	3	3	15
		50 m	3	3	-	3	3	3	15
		100 m	3	3	-	3	3	3	15
Up grade	No tree	0 m	3	3	3	3	3	3	18
		10 m	3	3	3	3	3	3	18
		30 m	3	3	3	3	3	3	18
		50 m	3	3	3	3	3	3	18
		100 m	3	3	3	3	3	3	18
	Tree	0 m	3	3	3	3	3	3	18
		10 m	3	3	3	3	3	3	18
		30 m	3	3	3	3	3	3	18
		50 m	3	3	3	3	3	3	18
		100 m	3	3	3	3	-	3	15
Total			60	60	45	57	57	63	342

### 2.4. Sample Processing

Each soil sample was air-dried in the laboratory, pulverized with an agate mortar and pestle, and then sieved through nylon sieve with diameter of ≤0.149 mm. 0.3 ± 0.0001 g sieved soil sample was then mixed with 6 mL HNO_3_–3 mL HCl–0.25 mL H_2_O_2_ in a tetrafluoroethylene (PTFE) beaker and heated in a microwave digestion system (GEM mars). After that, the digested solution was diluted to 50 mL with ultra-pure water and filtered through 0.45 μm microporous membrane. Finally, 1.0 mL filtered solution was diluted to 10 mL for measurement of Pb, Cd, Cu and Zn by Inductively Coupled Plasma-Mass Spectrometry (ICP-MS, Thermo X Series 2). Besides, a series of standard samples were also prepared. Quality controls involved: (1) analysis of 14 random samples, 1 blank samples and 1 national standard samples each time; and (2) random selection of samples to ensure that the relative standard deviation is less than ~10%.

## 3. Results and Discussion

### 3.1. Average Concentrations of Heavy Metals in Roadside Soils

The basic statistical descriptions of heavy metal concentrations (mg/kg) in roadside soils are listed in Table 3. On average, the concentrations of Cu (19.99 mg/kg), Zn (76.30 mg/kg), Cd (0.36 mg/kg), and Pb (22.57 mg/kg) in the mountainous rural farmland topsoil are lower than the tolerable levels, which are considered as phytotoxically excessive: 100 mg/kg of Cu, 300 mg/kg of Zn, 3 mg/kg of Cd, and 100 mg/kg of Pb [33]. However, the maximum Zn, Cd, and Pb concentrations in a few sites are close to or higher than the tolerable levels. The observations indicate that although the average accumulations of heavy metals pose no hazard in the region, some spots with peak concentrations may be severely polluted. For example, the maximum Pb concentration at the edge of highway with 800 m altitude, upgrade terrain, and no tree protection is up to two times higher than the tolerable level.

**Table 3:** Descriptive statistical results of heavy metal concentrations (mg/kg) in mountainous rural roadside farmland topsoil.

Variable	Level	N	Cu			Zn			Cd			Pb		
			Mean	S.D.	Max	Mean	S.D.	Max	Mean	S.D.	Max	Mean	S.D.	Max
Altitude	800 m	60	18.01	5.29	32.4	60.57	55.61	300	0.34	0.32	1.81	34.75	40.3	210
	1,000 m	60	22.17	4.68	30	97.63	57.12	436	0.54	0.72	4.97	30.15	24.83	146
	1,200 m	45	17.08	4.2	31	77.61	32.46	159	0.36	0.41	1.75	14.97	11.21	51.8
	1,400 m	57	13.73	6.05	31.3	38.78	20.17	120	0.35	0.39	1.87	10.4	8.57	40.2
	1,600 m	57	23.15	6.2	44.5	93.64	60.49	404	0.25	0.27	1.25	19.19	15.28	86
	1,800 m	63	24.66	7.26	45.8	88.27	48.14	272	0.3	0.45	3.07	23.26	15.22	83.2
Distance	0 m	66	19.75	7.11	45.8	69.52	37.85	179	0.38	0.37	1.75	28.83	37.16	210
	10 m	69	19.98	6.58	38	81.28	62.1	404	0.35	0.44	1.87	22.91	21.7	128
	30 m	69	20.53	7.11	44.5	81.74	59.23	436	0.36	0.4	1.81	22.23	19.62	94.3
	50 m	72	19.32	7.59	36.5	71.42	46.55	272	0.36	0.7	4.97	17.67	14.49	83.2
	100 m	66	20.39	6.14	31.9	77.49	53.35	308	0.33	0.28	1.14	21.67	20.04	95.8
Terrain	Down	165	19.77	6.98	45.8	85.26	63.28	436	0.34	0.4	3.07	18.23	13.97	86
	Up	177	20.18	6.85	41.4	67.94	38.36	230	0.37	0.51	4.97	26.62	29.71	210
Tree	No tree	180	22.05	7.61	45.8	83.18	56.9	436	0.34	0.41	3.07	23.71	27.91	210
	Tree	162	17.69	5.16	32.4	68.65	46.21	300	0.37	0.52	4.97	21.31	18.22	95.8
For All Samples		342	19.99	6.91	45.8	76.3	52.54	436	0.36	0.46	4.97	22.57	23.81	210

In order to assess roadside soil quality for heavy metals, pollution index (Pi) is calculated for comparing the heavy metal concentrations of roadside samples within 50 m distance (Ci) to the average concentrations of soil samples taken from 100 m for each altitude as the local background values (Xa). The pollution index (Pi) is calculated as Ci divided by Xa. Table 4 summarizes the descriptive statistical results of heavy metal concentrations comparison between roadside samples and local background values. On average, the Pi values of Cu, Zn, Cd, and Pb fluctuate around one, which do not indicate serious pollution owing to traffic emission. However, the maximum values of Pi are much higher than 1. The maximum roadside concentrations of Cu, Zn, Cd, and Pb are up to 1.99, 5.69, 12.52, and 7.75 times of the local background values. It is found that there is a clear trend that both average and maximum Pi values of Pb are decreasing with the roadside distance. Additionally, the Pi values of heavy metals in the samples without tree protection tend to be higher than those with tree protection, except for Cd.

**Table 4:** Descriptive statistical results of heavy metal concentrations comparison between roadside samples and local background values.

Variable	Level	Pi _Cu		Pi _Zn		Pi _Cd		Pi _Pb	
		Mean	Max	Mean	Max	Mean	Max	Mean	Max
Altitude	800 m	0.89	1.64	1.09	5.29	1.06	5.51	1.35	**7.75**
	1,000 m	0.94	1.20	1.34	**5.69**	1.46	**12.52**	1.19	5.57
	1,200 m	0.96	1.76	1.15	2.30	1.22	5.73	1.02	3.51
	1,400 m	0.83	1.87	1.16	3.48	1.17	6.15	1.14	4.28
	1,600 m	1.03	1.97	0.77	3.47	0.86	4.46	0.57	1.77
	1,800 m	1.09	**1.99**	0.68	2.28	0.80	8.65	1.01	3.62
Distance	0 m	0.94	**1.99**	0.91	2.30	1.13	5.73	1.21	**7.75**
	10 m	0.97	1.87	1.12	5.29	1.05	6.15	1.10	4.73
	30 m	1.00	1.97	1.15	**5.69**	1.10	4.67	1.04	3.51
	50 m	0.92	1.61	0.91	3.00	1.07	**12.52**	0.85	3.62
Terrain	Down	0.95	**1.99**	1.13	**5.69**	1.00	8.65	0.88	4.28
	Up	0.96	1.80	0.92	3.00	1.16	**12.52**	1.20	**7.75**
Tree	No tree	1.05	**1.99**	1.07	**5.69**	1.05	8.65	1.13	**7.75**
	Tree	0.86	1.76	0.96	5.29	1.13	**12.52**	0.96	3.51

**Table 5:** Correlation analysis of the dependent variables.

	Cu	Zn	Cd	Pb
Cu	1.000	0.375 **	0.092	0.259 **
Zn		1.000	0.202 **	0.217 **
Cd			1.000	0.307 **
Pb				1.000

** Correlation is significant at the 0.01 level (2-tailed).

Except for vehicle emissions, the concentrations of heavy metals in soil can be influenced by other local factors, such as the use of agricultural fertilizers and pesticides, climate and anthropogenic activities. The correlation analysis of the typical soil heavy metals associated with traffic activities will contribute to understand the homology of pollution source. As shown in Table 5, the correlation analysis of the dependent variables shows that either Cu or Cd content in roadside soil is significantly correlated with Zn and Pb content while there is no correlation between Cu and Cd. This finding can be reasonably explained by vehicular heavy metal emission processes. Almost 100% of Cu emission comes from brake wear while 83% of Cd emission comes from engine oil consumption [12]. However, Pb and Zn emissions are rather equally distributed in the mechanisms of fuel consumption, engine oil consumption, brake wear, or tire wear. The correlation pattern indicates that the heavy metal concentrations in roadside soils are associated with traffic contamination although the daily traffic volume in the Trishuli Highway is very low.

In the subsequent statistical analyses, a MANOVA is used to investigate differences between factors (see Table 6). The hypothesis testing in the following analysis is based on a 0.05 significance level. The result indicates that the independent factors are complicatedly associated with the concentrations of Cu, Zn, and Pb. The Cu concentration is significantly influenced by Altitude (*p* < 0.001), Tree (*p* < 0.001), and their two-way interaction (*p* < 0.001); the Zn concentration is significantly influenced by Altitude (*p* < 0.001), two-way interaction between Altitude and Tree (*p* < 0.001), and two-way interaction between Terrain and Tree (*p* = 0.025); and the Pb concentration is significantly influenced by Altitude (*p* < 0.001), Terrain (*p* < 0.001), Tree (*p* = 0.015), two-way interaction between Altitude and Distance (*p* = 0.012), two-way interaction between Altitude and Terrain (*p* < 0.001), two-way interaction between Altitude and Tree (*p* < 0.001), and two-way interaction between Tree and Distance (*p* = 0.004). However, except for Altitude (*p* = 0.039), no other factors have a significant effect on concentration of Cd. Meanwhile Cd is the least correlated with other heavy metals. Comparing the sums of correlation coefficient values among the heavy metals, Cd (0.601) is lower than Cu (0.726), Zn (0.794), and Pb (0.783), which suggests that the Cd distribution in Kathmandu region might be affected by some other resources. For example, the phosphorus fertilizer use can add heavy metals to farmland soil [34].

**Table 6:** MANOVA result for metal concentrations of Cu, Zn, Cd, and Pb.

Source	df	Cu		Zn		Cd		Pb	
		F	Sig.	F	Sig.	F	Sig.	F	Sig.
Altitude	5	23.916	0.000 **	6.525	0.000 **	2.378	0.039 *	11.127	0.000 **
Terrain	1	0.344	0.558	3.846	0.051	1.719	0.191	13.898	0.000 **
Tree	1	15.136	0.000 **	1.772	0.184	0.027	0.869	5.931	0.015 *
Distance	4	0.164	0.686	0.005	0.942	0.391	0.532	1.761	0.185
Altitude × Distance	20	1.878	0.098	2.193	0.055	0.433	0.826	2.966	0.012 *
Altitude × Terrain	5	1.790	0.115	0.508	0.770	2.171	0.057	10.100	0.000 **
Altitude × Tree	5	14.348	0.000 **	5.430	0.000 **	0.895	0.484	5.896	0.000 **
Terrain × Distance	4	3.683	0.056	1.172	0.280	0.657	0.418	3.827	0.051
Tree × Distance	4	1.413	0.236	0.158	0.691	0.089	0.766	8.423	0.004 *
Terrain × Tree	1	0.217	0.641	5.097	0.025 *	2.362	0.125	2.284	0.132

* Correlation is significant at the 0.05 level (2-tailed); ** Correlation is significant at the 0.01 level (2-tailed).

### 3.2. Concentrations of Heavy Metals Distribution by Altitude

**Figure 3:**
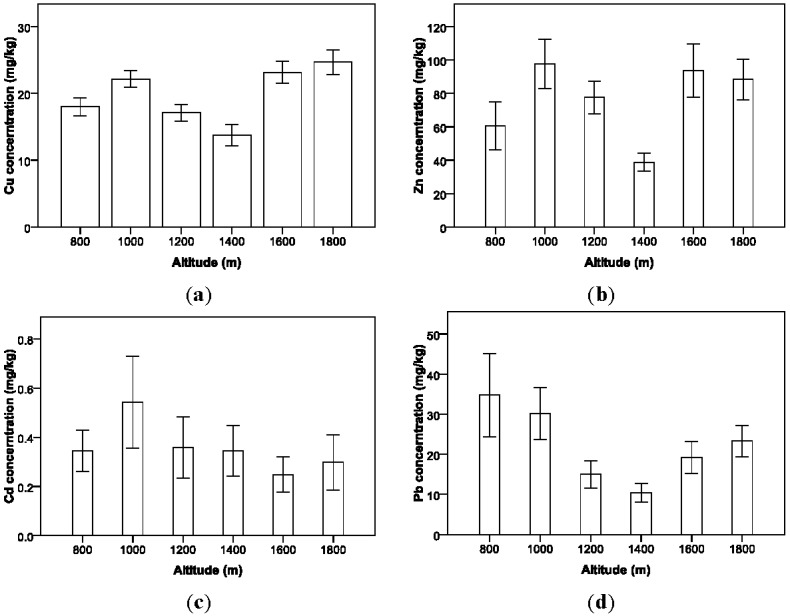
Variations in soil heavy metal concentrations with altitude.

The percentage of oxygen in the atmosphere decreasing with the increment of altitude can influence the efficiency of gas consumption and vehicular emission mechanism. Previous research focusing on the effect of altitude on vehicle on-road emissions indicated that vehicular emissions at high altitude can be much higher than observed at sea level [35,36,37]. As shown in Figure 3, among the six levels of altitude, the Cu concentrations at 1,400 m (M = 13.73 mg/kg; S.D. = 6.05 mg/kg) and 1,200 m (M = 17.08 mg/kg; S.D. = 4.20 mg/kg) are significantly lower than the other levels; the Zn concentration at 1,400 m (M = 38.78 mg/kg; S.D. = 20.17 mg/kg) is almost half of the other levels; and there is a similar trend for Pb distribution by altitude also. It should be noted that the highway segments for sampling at 1,400 m and 1,200 m have large elevation variations. Especially for the 1,400 m segment, it increases 70 m in height within a length of 1,000 m. Such a topographic feature can cause rainfall runoff to carry more heavy metal contaminants down to the lower highway segments or out of the highway through its drainage system. The results indicate that altitude is an important block factor in identifying the other factors effects. However, because the sampling region in this study is fully covered by vegetation, there is no significant difference in percentage of oxygen between the sampling locations with increasing altitudes. Therefore, the assumed pattern that the increasing altitudes may lead to consistently increasing roadside heavy metal concentration level cannot be captured in this study. 

### 3.3. Concentrations of Heavy Metals Influenced by Terrain

**Figure 4:**
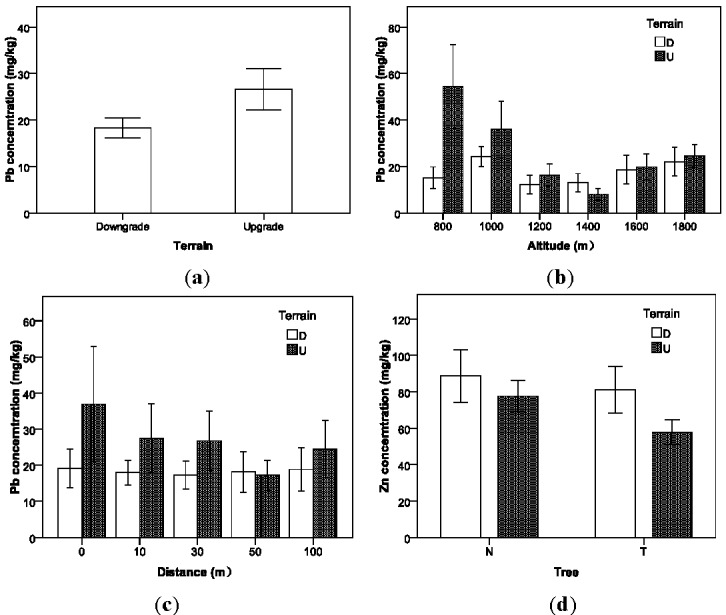
Variation in soil heavy metal concentrations with terrain.

Terrain displays a complex impact on Pb concentration in the soil but have no effect on the other metals, except that there is an interaction effect with Tree on Zn, as shown in Table 6. It was found that the Pb concentration in the downgrade roadside soil (M = 18.23 mg/kg; S.D. = 13.97 mg/kg) is significantly lower than that in the upgrade soil (M = 26.62 mg/kg; S.D. = 29.71 mg/kg), as shown in Figure 4(a). This finding is consistent with the previous conclusion that near roadways terrain morphology affects the distribution of Pb contamination and higher concentration values in the upgrade roadside soils as a consequence of obstructed dispersion [38]. This trend is especially significant at the altitude levels of 800 m, 1,000 m, and 1,200 m, as shown in Figure 4(b). The concentration of Pb in the top layer of soils differs between upgrade and downgrade farmland possibly because of the deposition and accumulation of atmospheric particulates from vehicle emission. Road emission was reported as the largest resource for Pb contamination for European countries and lead concentrations in topsoil are spatially heterogeneous [39]. The upgrade roadside soil would serve as a windward slope exposed to the deposition of atmospheric particulates generated from vehicle emission. This effect is further illustrated by Figure 4(c), which indicates that the Pb concentration decreases with the distance from road in the upgrade side of farmland, but it is uniformly distributed in the downgrade side. The rain runoff can also cause lead to transfer from further roadside soil to closer roadside soil since lead is immobilized by the soil [40]. 

On the contrary, Zn concentration in the downgrade roadside soil (M = 85.26 mg/kg; S.D. = 63.28 mg/kg) is marginally higher than that in the upgrade soil (M = 67.94 mg/kg; S.D. = 38.36 mg/kg), as shown in Table 6 (*p* = 0.051). Zn contaminants due to vehicle emissions are mainly from engine oil, tire wear, and brake wear [12], and their deposition to roadside soils are greatly influenced by the road surface runoff process. The road surface and roadside runoff process in mountainous terrain is significantly different from that in the flat highways [41]. Therefore, the rain runoff may carry more Zn contaminants to the downgrade farmland soil than the upgrade farmland soil. Intuitively, roadside trees can block more contaminants transferring the contaminants through water to the upgrade farmland, as shown in Figure 4(d).

### 3.4. Effect of Tree Protection on Heavy Metal Contamination

**Figure 5:**
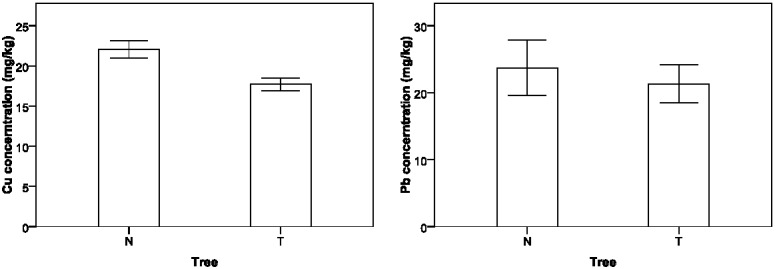
Variation in soil heavy metal concentrations with tree protection.

**Figure 6:**
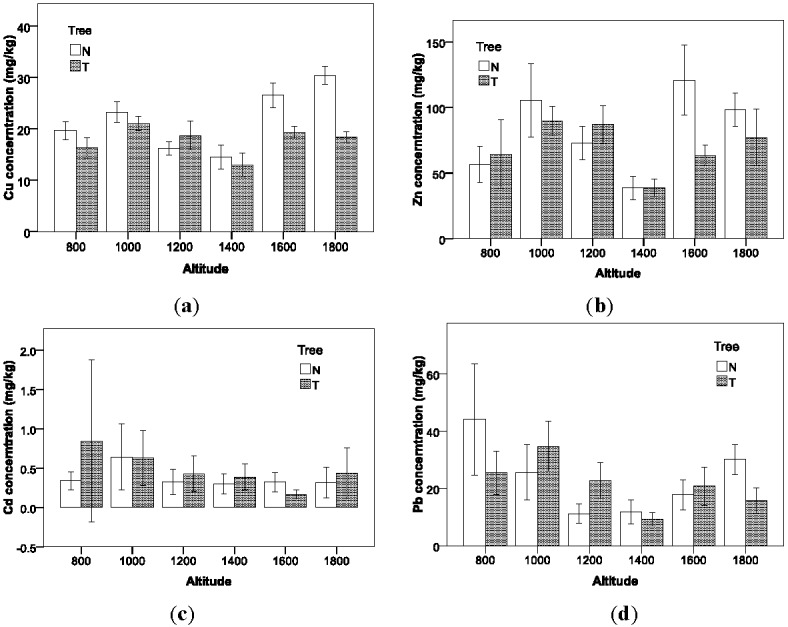
Interaction between altitude and tree.

It is found that roadside trees show a positive effect on the heavy metal concentration control, and concentrations of Cu and Pb in the roadside soils are statistically lower than those without tree protection, as shown in Figure 5. The interactive effects between altitude and tree for Cu, Zn, and Pb show that for some cases, the heavy metal concentrations with roadside trees may be equivalent to or higher than without trees, as shown in Figure 6, which might be caused by unknown local environmental factors that were not covered by this experiment. Planting trees can effectively prevent the pollution particles from depositing on roadside farmland so that more heavy metal contaminants can be expelled into drainage facilities. In addition, trees can be used to remove, transfer, or stabilize heavy metal soil contaminants to render them harmless [42]. In recent studies, phytoremediation has been considered as a promising new countermeasure for *in situ* cleanup of heavy-metal contaminated soils [43,44].

**Figure 7:**
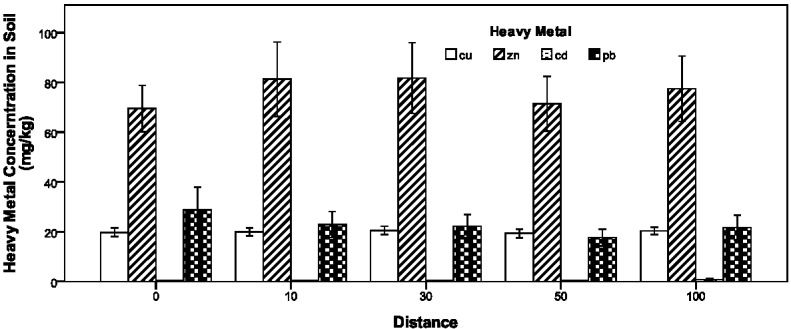
Variation in soil heavy metal concentrations with distance.

### 3.5. Effect of Distance to Road Edge on Heavy Metal Contamination

Generally, the heavy metal content in roadside soil has a belt-shaped distribution in terms of distance to road edge, decreasing exponentially with increment of roadside distance [26]. However, in this study such a distribution pattern by roadside distance is not available, as shown in Figure 7. Even graphically checking the samples site by site, no more than 20 percent of cases display a gradually decreasing distribution by roadside distance. The phenomenon can be explained by several reasons. Firstly, frequent farming activities such as irrigation, plough, and fertilization may mix the farmland top soil spatially and disturb the roadside heavy metal distance-distribution pattern. Secondly, the crops in roadside farmland may have various abilities to assimilate heavy metal soil contaminants. Thirdly, the complex local terrain and environments, such as rain runoff and drainage, wind direction and speed, and other non-crop plants might change the heavy metal contaminants’ distribution patterns in terms of roadside distance. Very few previous studies on roadside farmland soil contamination explored the relationship between heavy metal concentrations and roadside distance. According to the recent research focusing on Pb concentrations in roadside farmland soils, the Pb accumulation due to traffic activities is restricted to within 10 m of the motorway and 3 m of the minor road [45]. At distances greater than these, the other sources make the dominant contribution to the Pb concentration in farmland soil. Therefore, selecting 100 m as the maximum roadside sampling distance in this study would sufficiently cover the vehicle pollution’s scope. However, in nonagricultural land soils, the influential roadside distance of traffic pollution is generally less than 50 m but may be up to 100 m [27,28]. Even, Pb as the most adapted tracer of highway contamination may have an impact on roadside soil up to 320 m [13]. 

## 4. Conclusions

This study involving factorial design analyses was focused on heavy metal accumulation in roadside farmland soils along a highway across mountainous areas in Nepal. It was observed that although the average concentrations of Cu, Zn, Cd, and Pb at the sampling sites are lower than the tolerable levels, a few maximum Zn, Cd, and Pb concentrations are close to or higher than the tolerable level. It indicates that some roadside spots may be severely polluted. Furthermore, the correlation analysis showed that either Cu or Cd content is significantly correlated with Zn and Pb content but no significant correlation between Cu and Cd was identified. The pattern can be reasonably explained in terms of vehicle emission mechanism, which suggests the heavy metals’ homology of the traffic pollution source.

Due to the complex terrain characteristics of the representative environments, it is a challenging task for the experimental design to identify the most important factors associated with heavy-metal contamination from traffic activities. Based on a careful investigation on the roadside features of the Trishuli Highway, altitude, roadside distance, terrain, and tree protection are selected as four typical factors for this experiment. The MANOVA results indicate that the factors are complicatedly associated with the concentrations of Cu, Zn, and Pb, except for Cd. The heavy metal concentrations vary at the different altitude levels but display a similar variation pattern. Although altitude is not meaningful for explanation of heavy metal contamination, as a block factor it plays an important role in identifying the other factors effects. It was found that Pb concentration in the downgrade roadside soil is significantly lower than that in the upgrade soil, but Zn concentration in the downgrade roadside soil is marginally higher than that in the upgrade soil. This opposite trend might be due to the difference in deposition forms between Pb and Zn contaminants, where Pb is mainly transferred through air deposition while Zn is more likely to be carried through water runoff. 

The analysis indicates that trees growing linearly along roadways can effectively reduce the heavy metals’ concentrations in the roadside farmland. Therefore, planting trees may be considered as an effective countermeasure for existing crop plots that are close to roadways. Furthermore, in this study it was not found that the heavy-metal concentrations in the rural farmland soils are consistently decreasing with the increment of roadside distance. This conclusion differs from most of the finding in the previous urban roadside soil studies, primarily because of the lower traffic volume on the Trishuli Highway. On the other hand, the spatial distribution of heavy-metal contaminants in the roadside farmland topsoil could be disturbed by the frequent farming activities, crop growth distribution, and complex local terrain and environments. Therefore, it is suggested for the future roadside farmland studies to do more detailed sampling in the first 10 m from road edge for capturing the spatial heavy-metal distribution pattern. 

Finally, the findings of this study would be useful for understanding how the heavy-metal content in rural farmland roadside soil is influenced by traffic activities and helpful in making policies for avoiding hazardous heavy metal contaminants in agricultural soils in mountainous areas of the Third Pole region.
